# 

**Published:** 1882-05

**Authors:** 


### CONTENTS:

PAGE.

ORIGINAL COMMUNICATIONS:

Symptomatology of the Pupil. By Lucien Howe, M. D., . . . . . . 433

CLINICAL REPORTS:

Case Illustrating the Advantages of Median Lithotomy. Reported by George E. Brewer, . . . . . . . . 440

TRANSLATIONS:

Remarks on Syphilitic Affections of the Eye—Extract from a Lecture by Prof. L. de Wecker. From the French by Lucien Howe, M. D., . . . . . . . 443

SELECTIONS:

The Pathology of Malaria, . . . . . 446

Treatment of Goitre with Ergotin, . . 448

Sanitary Relations of the Soil. By Dr. Max Von Pettenkofer, . . . . . . 450

Unpleasant Results from the Use of Iodoform, . . . . . . . . . 461

Dilatation of the Cervical Canal for Spasmodic Dysmenorrhœa and Sterility, . 463

The Formation of Blood-Corpuscles, . . 465

Pernicious Anæmia, . . . . . 468

Post-Partum Hæmorrhage, . . . . 470

The Artificial Production of Organic Forms, 471

Incubation in Various Diseases, . . . 472

SOCIETY REPORTS:

Buffalo Medical and Surgical Association, . 473

EDITORIAL, . . . . . . . . 474

Editorial Notes, . . . . . . . 476

REVIEWS, . . . . . . . . 477

